# Using Self-Regulated Learning Microanalysis to Examine Regulatory Processes in Clerkship Students Engaged in Practice Questions

**DOI:** 10.5334/pme.833

**Published:** 2023-10-13

**Authors:** Mary A. Andrews, Catherine A. Okuliar, Sean A. Whelton, Allison O. Windels, Stacy R. Kruse, Manesh G. Nachnani, Deborah A. Topol, Elexis C. McBee, Michael T. Stein, Raj C. Singaraju, Sam W. Gao, David S. Oliver, Jed P. Mangal, Jeffrey S. LaRochelle, William F. Kelly, Kent J. DeZee, H. Carrie Chen, Anthony R. Artino, Paul A. Hemmer, Ting Dong, Timothy J. Cleary, Steven J. Durning

**Affiliations:** 1 Department of Medicine, F. Edward Hébert School of Medicine, Uniformed Services University, Bethesda, Maryland, USA; 2 Department of Medicine, Georgetown University School of Medicine, Washington, District of Columbia, USA; 3 Department of Medical Education, University of Central Florida College of Medicine, Orlando, Florida, USA; 4 Department of Pediatrics, Georgetown University School of Medicine, Washington, District of Columbia, USA; 5 Department of Health, Human Function, and Rehabilitation Sciences, School of Medicine and Health Sciences, The George Washington University, Washington, District of Columbia, USA; 6 Center for Health Professions Education, F. Edward Hébert School of Medicine, Uniformed Services University, Bethesda, Maryland, USA; 7 Department of School Psychology, Graduate School of Applied and Professional Psychology, Rutgers, The State University of New Jersey, Piscatawy, New Jersey, USA

## Abstract

**Introduction:**

Self-regulated learning is a cyclical process of forethought, performance, and self-reflection that has been used as an assessment tool in medical education. No prior studies have evaluated SRL processes for answering multiple-choice questions (MCQs) and most evaluated one or two iterations of a non-MCQ task. SRL assessment during MCQs may elucidate reasons why learners are successful or not on these questions that are encountered repeatedly during medical education.

**Methods:**

Internal medicine clerkship students at three institutions participated in a SRL microanalytic protocol that targeted strategic planning, metacognitive monitoring, causal attributions, and adaptive inferences across seven MCQs. Responses were transcribed and coded according to previously published methods for microanalytic protocols.

**Results:**

Forty-four students participated. In the forethought phase, students commonly endorsed prioritizing relevant features as their diagnostic strategy (n = 20, 45%) but few mentioned higher-order diagnostic reasoning processes such as integrating clinical information (n = 5, 11%) or comparing/contrasting diagnoses (n = 0, 0%). However, in the performance phase, students’ metacognitive processes included high frequencies of integration (n = 38, 86%) and comparing/contrasting (n = 24, 55%). In the self-reflection phase, 93% (n = 41) of students faulted their management reasoning and 84% (n = 37) made negative references to their abilities. Less than 10% (n = 4) of students indicated that they would adapt their diagnostic reasoning process for these questions.

**Discussion:**

This study describes in detail student self-regulatory processes during MCQs. We found that students engaged in higher-order diagnostic reasoning processes but were not explicit about it and seldom reflected critically on these processes after selecting an incorrect answer. Self-reflections focused almost exclusively on management reasoning and negative references to abilities which may decrease self-efficacy. Encouraging students to identify and evaluate diagnostic reasoning processes and make attributions to controllable factors may improve performance.

## Introduction

Learners who do not achieve proficiency at the expected level of training are a common challenge encountered by medical educators [1,2,3]. Such learners often perform poorly on medical knowledge tests, which predicts poor performance on subsequent tests in medical training and may result in review by a promotions committee [3,4,5]. The personal and professional consequences of failing one or more of these examinations can be serious and include adverse impacts on self-esteem and self-confidence, extensions in training, delay or failure in obtaining licensure, and difficulty obtaining subsequent training or employment [6].

Large-scale, multicenter studies of ways to help learners who underperform on medical knowledge tests are lacking and therefore most test-taking remediation is guided by expert opinion and institutional experience. Methods for improving test performance reported in the literature include diverse activities such as directed reading programs, completion of assigned practice questions and/or progress tests, problem-based learning, mandatory attendance at didactic conferences, neuropsychological testing, and regular meetings with faculty members [7,8,9,10]. However, despite expert recommendations that remediation strategies be tailored to the individual learner, many remediation strategies for improving test performance represent a one-size-fits-all approach, where all learners who fail or underperform on a test are encouraged or required to engage in certain types of educational activities that may or may not include individualized assessment. Nevertheless, an important aspect of helping learners improve their performance is uncovering aspects of their strategic and regulatory approaches to learning and taking test questions. As a preliminary step towards this goal, the purpose of this multicenter study was to identify the nature and quality of various self-regulated learning (SRL) processes (i.e., strategic planning, metacognitive monitoring, causal attributions, and adaptive inferences) of clerkship students engaged in answering test questions.

### Theoretical Framework

**Figure 1:**
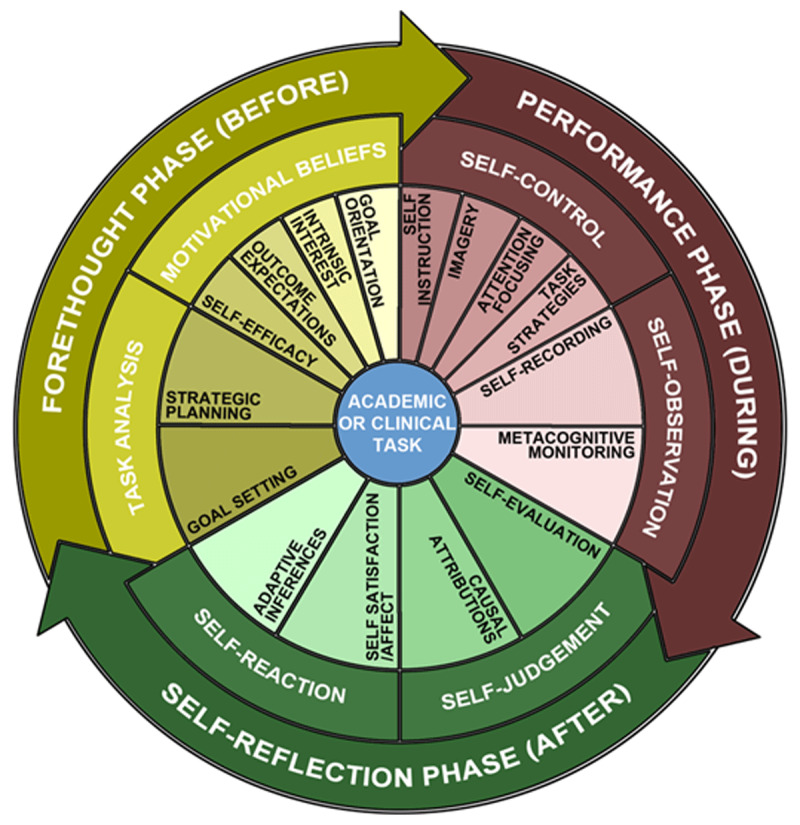
A three-phase, cyclical model of self-regulated learning (SRL). The model depicts three sequential phases of SRL: forethought (before), performance (during), and self-reflection (after). The model also shows, within each phase, the sub-processes of SRL. Adapted, with permission, from Artino AR, Jr., Jones KD. AM last page: self-regulated learning–a dynamic, cyclical perspective. Acad Med. Jul 2013;88(7):1048. Doi: 10.1097/ACM.0b013e3182953763.

SRL theory may provide a useful framework for conducting an individualized assessment aimed at identifying and remediating the deficits of underperforming learners [11]. SRL is often described as a triphasic cycle of forethought, performance, and self-reflection and has been used to understand the differences between expert and novice performers [12]. The three phases of SRL contain multiple subprocesses; for example, the forethought phase includes task analysis and strategic planning, the performance phase includes attention-focusing and metacognitive monitoring, and the self-reflection phase includes causal attributions and adaptive inferences (Figure 1) [13].

The focus of SRL processes often differ depending on the specific task at hand; for example, the strategies and metacognitive cues differ among a volleyball server, a free throw shooter, and a medical student trying to answer a practice question. Nevertheless, skillful self-regulation characterizes higher levels of human performance across a wide array of disciplines [12,14,15]. In studies of medical students that utilized SRL microanalysis, a technique which targets specific SRL subprocesses by means of open-ended questions in real-time during the three phases of an authentic task, researchers have found positive correlations between SRL processes such as strategic planning and educational outcomes such as learning task performance, course grade, and USMLE Step 1 scores [17]. By generating information about specific regulatory processes of learners while engaged in a given task, SRL microanalysis may assist faculty in determining why a student is underperforming on that task and thus inform the remediation plan.

Prior studies have utilized SRL microanalysis to describe the regulatory processes of medical learners engaged in preclinical neuroscience learning tasks [18], written and simulation-based clinical reasoning exercises [17,19,20], and procedural simulation [21]. The studies involving written clinical reasoning exercises in preclerkship students examined forethought and performance phase processes. In particular, for students engaged in a paper case, this work found that process-oriented diagnostic strategies (e.g., identifying key symptoms, integrating clinical information, and comparing/contrasting diagnoses) were positively associated with clinical reasoning course grade [17]. Furthermore, researchers found that over two iterations of a challenging clinical reasoning exercise, the quality of students’ strategic planning (forethought phase) and metacognition (performance phase) tended to decline after an initial incorrect attempt at arriving at the diagnosis, as students began to focus less on the diagnostic process and more on irrelevant aspects of the case [20]. Notably, this study did not investigate SRL processes in the self-reflection phase, which is a critical time for struggling students to evaluate their performance, determine where they went wrong, and decide on necessary adaptations. The study involving simulation-based clinical reasoning conducted in resident and staff physicians did examine self-reflection phase processes and found that only 3% (1/38) of physicians reflected critically on their diagnostic reasoning process after the exercise [19]. However, neither of these studies examined all three SRL phases (forethought, performance, and self-reflection). In addition, the tasks (simulation, paper clinical reasoning cases) differ from answering clinical vignette-based test questions in important ways (for example, having multiple-choice response format). Finally, the prior studies examined at most two iterations of a task, giving participants few opportunities to demonstrate their SRL capabilities. To describe all three SRL phases across multiple iterations for the task of answering clinical vignette-based multiple-choice questions (MCQs), we used SRL microanalysis to describe the SRL processes of lower-performing students as they engaged in a series of practice questions.

## Methods

Medical students at Uniformed Services University of the Health Sciences F. Edward Hébert School of Medicine, Georgetown University School of Medicine, and University of Central Florida College of Medicine were invited to participate. We chose to study clerkship students instead of preclerkship students because the clerkship provided a more uniform experience across the three institutions with 8-12 weeks of clinical internal medicine followed by the National Board of Medical Examiners Subject Examination in Medicine (NBME SEM). The study was initially designed as a randomized trial to test the effectiveness of a previously described intervention to improve test performance, with primary outcome of NBME SEM score as a primary outcome and secondary outcome of differences in self-regulatory processes during MCQs [22]. No significant group differences were found in academic outcomes or self-regulatory processes; thus, the intervention and control groups were collapsed into a single cohort for purposes of describing the regulatory processes of clerkship students during MCQs. The Institutional Review Boards at each participating institution approved this study.

### Eligibility and Description of Study Population

All students beginning the internal medicine clerkship at each site were invited to participate. Students were in their second or third year of medical school and entered the clerkship between September 2018 and December 2020. The preclerkship curricula at each institution were not identical but all complied with the standards of the Liaison Committee on Medical Education. As we sought to describe the SRL processes of students who were not the highest performers in medical school, participants were ineligible if they scored more than one standard deviation above the mean of the clerkship cohort (group of students starting the clerkship at the same time) on the NBME Self-Assessment in Medicine. To describe the study population, we collected participant age, gender, MCAT score, and NBME SEM score and percentile rank.

### Microanalytic Protocol

To describe the regulatory processes of students engaged in answering MCQs, we developed an SRL microanalytic protocol according to methods previously described and administered to study participants at the end of the internal medicine clerkship [22]. The protocol consisted of a scripted set of microanalytic questions asked at specific time points as the student engaged in a think-aloud through a clinical vignette-based MCQ. Thinking aloud was necessary to allow consistent timing of the microanalytic questions at specific points during the MCQ. These microanalytic questions were repeated over the course of seven MCQs (Table 1), were designed to assess the participant’s strategic planning (forethought phase), metacognitive monitoring (performance phase), and causal attributions and adaptive inferences (self-reflection phase), and were adapted from prior studies describing the application of SRL microanalysis to a clinical reasoning task [17,19,23]. These particular subprocesses were chosen based on prior experience utilizing SRL microanalysis to assist learners who struggle on multiple choice questions [22]. During the protocol each student was asked about each subprocess seven times throughout the exercise (once for each practice question). Student responses to these questions were audio-recorded and later transcribed. A full description of the SRL microanalytic protocol is available in the Supplement.

**Table 1:** Description of Self-Regulated Learning Subprocesses, Microanalytic Questions, Timing of Inquiry, and Examples of Coding Scheme Categories.


SRL SUBPROCESS	MICROANALYTIC QUESTION	TIMING	EXAMPLES OF CODING SCHEME CATEGORIES

Strategic Planning	“What are your plans for how to approach this question in order to answer it correctly?”	Before the learner begins the practice question	Diagnostic reasoning process Management reasoning process Test-taking tactics

Metacognitive Monitoring	“What have you been thinking about as you have been working through this test question?”	After the learner has read through the clinical vignette one time	Diagnostic reasoning process Management reasoning process Test-taking tactics Prior clinical experience Ability/knowledge

Causal Attributions	“What do you think is the number one reason why you got this question wrong?”	After the learner had selected an answer and been told the answer was incorrect	Diagnostic reasoning process Management reasoning process Prior clinical experience Ability/knowledge

Adaptive Inferences	“What do you need to do in order to get this question or a similar question right in the future?”	After the learner had been asked about causal attributions above	Diagnostic reasoning process Management reasoning process Test-taking tactics Increase knowledge


To categorize the responses, two authors (MA and JM), with guidance from a third author (TC), developed a coding scheme based on a prior SRL microanalytic protocol used to assess medical students engaged in a clinical reasoning task [18]. MA and JM initially used this rubric to code responses from six participants followed by discussion and revising of the coding scheme. After finalizing the coding scheme, MA and JM independently coded responses from 5% of the total sample. A kappa coefficient was obtained for each of the four SRL subprocesses, revealing consistently strong levels of agreement: strategic planning (0.82); metacognitive monitoring (0.78); causal attributions (0.86); and adaptive inferences (0.76). The remainder of the sample was divided between the two authors and coded independently, with regular meetings to ensure observation of no new themes (thematic saturation) and to address questions which were resolved by discussion or by a third author (SD).

The final coding scheme included categories for diagnostic reasoning, management reasoning, test-taking tactics, personal preferences, outcome-oriented statements, reliance on prior experience, case difficulty, luck, focus/effort, personal interest in topic/case, ability/knowledge, don’t know, and other. Diagnostic reasoning was subcategorized as basic diagnostic reasoning (identifying symptoms/signs, identifying contextual factors, and prioritizing symptoms/signs) and advanced diagnostic reasoning (integrating symptoms/signs and comparing/contrasting diagnoses). Management reasoning was defined as responses reflecting the need to identify the next best diagnostic test to order or therapy to initiate. Test-taking tactics were defined as responses referencing methods, approaches, or procedures for understanding and completing the test question and primarily pertain to actions or a plan for working through the test question as a test question rather than a diagnostic reasoning exercise (e.g., read the question first, read the answers first, process of elimination, etc.).

A full description of the coding scheme and definitions of categories is available in the Supplement. The frequency of codes for each SRL subprocess were tabulated and expressed as the number and percent of participants who gave a response corresponding to that code at least once over the seven practice questions. Given the importance of cognitive flexibility for success in medical school and higher education settings, we also calculated the proportion of students who changed their strategic approach after a failed attempt at a practice question and the proportion who maintained the same strategy in response to failure [24,25].

## Results

**Figure 2:**
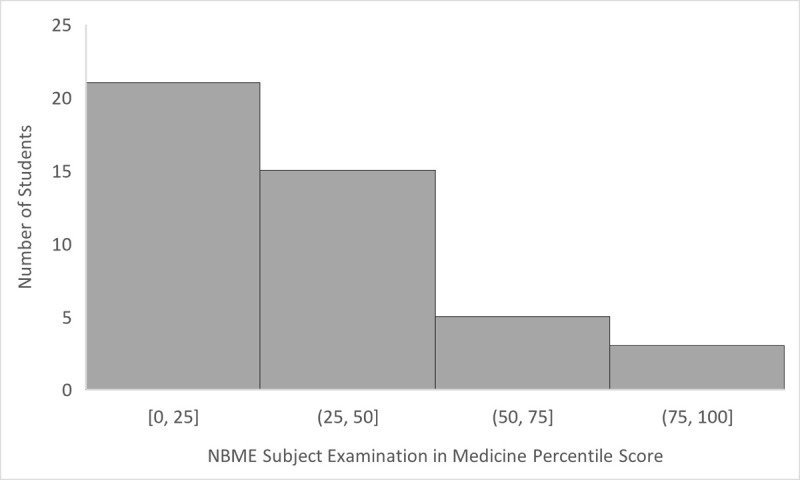
Distribution of National Board of Medical Examiners Subject Examination in Medicine Percentile Scores for Study Participants. Square brackets are inclusive of the endpoint.

Data on SRL microanalytic processes were gathered from 44 internal medicine clerkship students at three medical schools. Participants demographics are described in Table 2 and distribution of NBME SEM scores are shown in Figure 2.

**Table 2:** Demographic and Academic Characteristics of 44 Students Participating in a Self-Regulated Learning Microanalytic Assessment During Practice Questions.


CHARACTERISTIC	VALUE

Age, mean (SD)	26.9 (3.3)

Gender, n (%)	

Male	17 (39)

Female	27 (61)

MCAT, mean (SD)	509 (6)

Site, n (%)	

USU	24 (54)

GU	17 (39)

UCF	3 (9)

NBME Subject Examination in Medicine, mean score (SD)	69.7 (8.6)


Abbreviations: MCAT = Medical College Admission Test; NBME = National Board of Medical Examiners.

The frequencies of response categories for four microanalytic processes of strategic planning, metacognitive monitoring, causal attributions, and adaptive inferences are shown in Tables 3, 4, 5, 6. Below we discuss the response frequencies with examples for each subprocess.

**Table 3:** Frequency of Responses to Microanalytic Questions about Strategic Planning for 44 Clerkship Students Engaged in Clinical Vignette-Based Multiple-Choice Questions.


RESPONSE CATEGORY	SUBCATEGORY	N	%*

Diagnostic Reasoning Process	Identify symptoms/signs	4	9

	Identify contextual/environmental factors	3	7

	Prioritize relevant features	20	45

	Integration of clinical information	5	11

	Comparing/contrasting diagnoses	0	0

	General/other diagnostic process	15	34

Management Reasoning Process		5	11

Test-taking Tactics	Read clinical vignette first	25	57

	Read prompt (question) first	26	59

	Read answers first	4	9

	Process of elimination	6	14

	Test-taking tactic not defined above	12	27

Personal Preferences		1	2

Prior Clinical Experience		1	2

Ability/Knowledge	Positive reference to ability/knowledge	0	0

	Negative reference to ability/knowledge	1	2

Don’t Know		2	5

Other		11	25


* Students had seven opportunities to offer responses and responses often included elements from more than one category. Thus, column totals do not equal the total number of students and percentages do not sum to 100.

### Strategic Planning

Regarding strategic planning for the MCQ (Table 3), between 7–45% of students mentioned the relatively simple diagnostic reasoning strategies of identifying symptoms/signs, identifying contextual factors, or prioritizing relevant features. An example of the prioritization category is the response, “I like to kind of underline or highlight the key things that I think are important,” and “I immediately start, when I look through it, I look for the keywords and start making a differential almost right as I start reading it.” Many students (n = 15, 34%) mentioned nonspecific diagnostic strategies that could not be further subclassified and which commonly reflected a desire to find the right diagnosis without a more detailed plan for accomplishing that. For example, one student said, “I’m going to read the question, think about the diagnosis while I’m reading the question, and pick the diagnosis before I pick the answer choice.” Few students mentioned the higher-order diagnostic reasoning processes of integrating clinical information (n = 5, 11%) or comparing/contrasting diagnoses (n = 0, 0%) as their strategic focus. Some students (n = 11, 25%) gave strategy responses difficult to categorize and counted under “Other” such as the student who indicated that part of his strategy was, “Just kind of see where my mind takes me as it goes.” Five (11%) gave responses pertaining to management reasoning, while in terms of test-taking tactics, about even numbers of students responded at least once during the seven practice questions that they were going to read the clinical vignette first (n = 25, 57%) and read the prompt first (n = 26, 59%). Fifteen of 41 students (37%) changed their strategy in response to an incorrect answer to a practice question, while n = 26 (63%) maintained the same strategy.

### Metacognition

In response to a question about metacognitive monitoring, or what the students were thinking about as they completed the test question, students continued to use basic diagnostic strategies such as identifying symptoms/signs (n = 11, 25%), identifying contextual factors (n = 16, 36%), and prioritizing relevant features (n = 18, 41%). However, they were much more engaged with higher-level diagnostic reasoning processes than they had initially indicated when asked about their strategic planning before the question. While only 11% (n = 5) of students mentioned integrating clinical information as their strategic focus, 38 (86%) students gave metacognitive responses indicating that they were indeed engaged in integrating the clinical information as they worked through the practice question. As an example of a response indicating attempts at integration of clinical information as the primary metacognitive focus, one student noted, “[The patient] also has an elevated total bilirubin and elevated creatinine. Those two things in a patient with that history and elevated ammonia are already pointing me in the direction of a decompensated cirrhosis picture.” Likewise, while no students mentioned comparing/contrasting diagnoses as their chosen strategy, 24 (55%) students were indeed engaged in this technique based on their responses to the metacognitive monitoring question. (Table 4) An example of the comparing/contrasting diagnoses category was the response, “His leukocyte count is 9,000 with lymphocyte predominance. His platelets are a little bit low. Peripheral blood smear shows large atypical lymphocytes… I was thinking some sort of subacute viral type of illness versus a lymphoma or leukemia.”

**Table 4:** Frequency of Responses to Microanalytic Questions about Metacognitive Monitoring for 44 Clerkship Students Engaged in Clinical Vignette-Based Multiple-Choice Questions.


RESPONSE CATEGORY	SUBCATEGORY	N	%*

Diagnostic Reasoning Process	Identify symptoms/signs	11	25

	Identify contextual/environmental factors	16	36

	Prioritize relevant features	18	41

	Integration of clinical information	38	86

	Comparing/contrasting diagnoses	24	55

	General/other diagnostic process	32	73

Management Reasoning Process		34	77

Test-taking Tactics	Read clinical vignette first	0	0

	Read prompt (question) first	1	2

	Read answers first	0	0

	Process of elimination	4	9

	Test-taking tactic not defined above	6	14

Personal Preferences		1	2

Prior Clinical Experience		3	7

Ability/Knowledge	Positive reference to ability/knowledge	2	5

	Negative reference to ability/knowledge	10	23

Don’t Know		3	7

Other		8	18


* Students had seven opportunities to offer responses and responses often included elements from more than one category and thus column totals do not equal the number of students in each group nor do percentages sum to 100.

Many student responses to the metacognitive monitoring question were coded in the management reasoning category (n = 34, 77%). As an example of management reasoning, one student noted, when working through a problem about a patient with COPD presenting with failure of outpatient management, “Giving her six times a day of short-acting inhaler doesn’t sound like the way to solve this problem.” Another student, for the same vignette, noted, “She has an oxygen requirement, pretty significantly, with an unstable respiratory rate. So management-wise, thinking not sending her home.” Occasionally, students’ personal preferences for or against certain topics would emerge in their metacognition, such as when a student commented, “I hate pulmonary… absolutely hate it,” when asked what they were thinking about during the practice question on COPD. Additionally, 10 (23%) student responses were focused on negative references to their own knowledge or ability.

### Causal Attributions

Regarding causal attributions, which were queried whenever students answered a MCQ incorrectly, students rarely pointed to defects in their selected diagnosis or diagnostic reasoning process as the reason for their failure to answer a question correctly (1-9% across all diagnostic reasoning categories, Table 5). In contrast, students very often (n = 41, 93%) focused on their management reasoning process as the reason they answered the question incorrectly. For example, one student said, “I think I know from somewhere that you’re supposed to do max doses of one medication before you add something else, so that’s probably just something I should have remembered.” Another student identified the problem with their management reasoning in this way: “If you have a UTI or pyelonephritis that doesn’t respond, usually you think about an abscess, and I could have just applied that to this [patient with prostatitis].” Sometimes participants referred to prior clinical experiences to explain their incorrect answer choice, such as a student who said, “I’ve only ever given lactulose for this and I’ve never had it not work,” and another who said, “I haven’t seen really many patients with COPD, so I guess I just didn’t have the foundation.” Some students attributed their incorrect answer to prior clinical experience, where what they observed in actual clinical practice did not correspond to practice question (n = 18, 41%). Many students (made negative references to their knowledge or abilities (n = 37, 84%) as the primary reason for their poor performance.

**Table 5:** Frequency of Responses to Microanalytic Questions about Causal Attributions for 44 Clerkship Students Engaged in Clinical Vignette-Based Multiple-Choice Questions.


RESPONSE CATEGORY	SUBCATEGORY	N	%*

Diagnostic Reasoning Process	Identify symptoms/signs	1	2

	Identify contextual/environmental factors	1	2

	Prioritize relevant features	4	9

	Integration of clinical information	2	5

	Comparing/contrasting diagnoses	2	5

	General/other diagnostic process	2	5

Management Reasoning Process		41	93

Test-taking Tactics	Read clinical vignette first	0	0

	Read prompt (question) first	0	0

	Read answers first	0	0

	Process of elimination	5	11

	Test-taking tactic not defined above	5	11

Personal Preferences		5	11

Prior Clinical Experience		18	41

Ability/Knowledge		37	84

Question difficulty		2	5

Question was unfair/defective		2	5

Don’t Know		0	0

Other		9	20


* Students had up to seven opportunities to respond and student responses often included elements from more than one category and thus column totals do not equal the number of students nor do percentages sum to 100.

### Adaptive Inferences

When asked about their adaptive inferences, which are the conclusions that students made about what they needed to do to answer a similar question correctly in the future, students again very seldom pointed to changes in their diagnostic reasoning processes (0-9% across all categories, Table 6). A large percentage of the responses reflected changes or improvements in management reasoning processes (n = 26, 59%), such as, “I think just go through the sort of tree of management for COPD,” and another who said, “[I need to] figure out how to treat different arrhythmias.” Students seldom reflected critically on test-taking tactics, with no students determining that they needed to change the order in which they approached the test question. Students often identified reading more as their plan for improvement after answering a question wrong (n = 20, 45%), with few details about how they would implement this plan. For example, one student’s plan was “look over C. diff complications and indications for surgery,” and another student’s response was, “review that [COPD treatment algorithm] again.” A somewhat more detailed response was, “So once again, go to Step-up to Medicine. I would go review over this complication of cirrhosis. I feel like, relatively quickly, I’d be able to identify what this was and then further delve into signs, symptoms, and management of this condition.” Other students identified a need to see more patients with a given condition or do more practice questions on that topic (n = 14, 32%). An example of this was a student who indicated that what they should do was “just [have more] experience, either real life or with practice questions or something.” Another student said what they should do was “probably work with patients who are on this medication and Google it as we speak now.”

**Table 6:** Frequency of Responses to Microanalytic Questions about Adaptive Inferences for 44 Clerkship Students Engaged in Clinical Vignette-Based Multiple-Choice Questions.


RESPONSE CATEGORY	SUBCATEGORY	N	%*

Diagnostic Reasoning Process	Identify symptoms/signs	0	0

	Identify contextual/environmental factors	1	2

	Prioritize relevant features	3	7

	Integration of clinical information	4	9

	Comparing/contrasting diagnoses	0	0

	General/other diagnostic process	2	5

Management Reasoning Process		26	59

Test-taking Tactics	Read clinical vignette first	0	0

	Read prompt (question) first	0	0

	Read answers first	0	0

	Process of elimination	3	7

	Test-taking tactic not defined above	8	18

Personal Preferences		6	14

Prior Clinical Experience		0	0

Focus/effort		3	7

Increase Knowledge/Ability	By reading on this topic	20	45

	By method other than reading (e.g., clinical exposure, practice questions)	14	32

	By method not specified	24	55

Don’t Know		1	2

Other		10	23


* Students had up to seven opportunities to respond and student responses often included elements from more than one category and thus column totals do not equal the number of students nor do percentages sum to 100.

## Discussion

In this study, we examined the regulatory processes of internal medicine clerkship students by targeting strategic planning (forethought phase), metacognition (performance phase), and causal attributions and adaptive inferences (self-reflection phase) during the task of answering clinical vignette-style practice MCQs, a skill that is critical for achieving licensure and certification but as yet has not been examined using SRL microanalysis. In a study that allowed multiple opportunities to observe SRL processes in individual participants, a key finding was discordance regarding what students say they are focused on before a practice question, what they actually do in terms of diagnostic reasoning during a practice question, and what they reflect critically upon afterwards. Further, there was a subset of students who focused primarily on their poor skills and abilities during the task completing the activity, with an extremely large percentage of students (n = 37, 84%) noting that their poor skills and abilities were the primary reason for their struggles. When medical educators can gain access to these types of student thoughts, strategic actions, and reactions to learning, they may be better equipped to provide feedback and coaching supports to these students [23].

When examining each of the four regulatory processes in depth, interesting findings emerged. The strategic planning of students before they engaged in the practice questions included relatively few higher-order diagnostic reasoning strategies such as integration of clinical information or comparing/contrasting diagnoses, although students did identify simpler diagnostic reasoning strategies such as identifying symptoms/signs and prioritizing relevant features. However, even though higher order diagnostic processes were not an explicit focal point during the forethought phase, it became apparent during the performance phase when asked about metacognition that students are indeed engaged in integration and comparing/contrasting at a relatively high frequency. These findings suggest that the students in this sample did not proactively and intentionally think about the key processes that they would ultimately use during the diagnostic reasoning process. When medical students approach learning or performance activities from a reactive rather than proactive perspective, they will likely be less efficient in completing activities and will rely on social or normative comparisons to evaluate themselves rather than their personal progress [12].

When it came to the self-reflection phase and students were asked about why they missed an answer (causal attributions) or what they needed to do better next time (adaptive inferences), students focused almost exclusively on management reasoning, while mentioning changes to their diagnostic approach less than 10% of the time. Students were relatively inflexible in their strategic approach, with most students maintaining the same strategy after making a mistake. Furthermore, nearly a quarter of student responses to the metacognitive monitoring question and 84% of attribution responses reflected negative thoughts about student’s own knowledge and abilities – a potentially distracting and maladaptive occurrence. Prior research has shown a relationship between attributing failures to factors outside student control (such as innate abilities) and declines in motivation and performance [28]. Outside of a microanalytic protocol, these negative thoughts may be completely hidden from the medical educator, which speaks to the importance of SRL microanalysis as an assessment tool. Knowing that a student is making negative reflections on their abilities, the educator can redirect them to consider factors within their control, such as their strategic approach to the question.

Ours is the first study using SRL microanalysis to examine medical student regulatory processes during clinical vignette-based MCQs, a task that students must master to achieve licensure and certification. In addition, this is one of few SRL microanalytic studies across human performance domains that examined all three phases of SRL [16,21]. In fact, the disconnect between what students say they will focus on, what they actually do, and what they reflect critically upon after completing a practice question would not be evident without examining all three phases of SRL. Given the cyclical, iterative nature of SRL and the insights gained from comparing intended strategy, executed strategy, and self-reflection on both, future studies utilizing SRL microanalysis may wish to routinely include questions targeting all three phases of SRL.

Our results confirm some aspects of prior research and suggest potential targets for improving the quality of medical students’ strategic planning, causal attributions, and adaptative inferences. A prior study using SRL microanalysis in medical students working through a paper case also described a relative paucity of higher level diagnostic strategies in the planning phase, with only 15% (11/71) students indicating integration as a their strategy [17]. Similar to our study, this study also found that even though students did not explicitly mention advanced diagnostic reasoning processes as their strategic focus before the exercise, the metacognitive monitoring responses indicate that they indeed are engaged in the diagnostic reasoning process, with 54% (38/71) of students doing integration during the exercise. Perhaps not surprisingly given that diagnostic reasoning was not an explicit strategic focus in the forethought phase, diagnostic reasoning also was not often identified as the reason for an incorrect answer nor mentioned as a target that the students would seek to change or improve. This corresponds to a prior study examining the self-reflection phase processes of physicians after a clinical reasoning exercise, in which only 3% of physicians (1/38) reflected on some aspect of their diagnostic reasoning process after a clinical reasoning exercise [19]. One might argue that a student may not identify the appropriate strategy in one or two paper clinical reasoning cases or simulation encounters, but it is notable that our study allowed students seven opportunities to identify and reflect on their strategy and yet we still found that only rarely do students cite higher-level diagnostic reasoning processes as a strategic focus or an area for improvement. Interventions aimed at encouraging students and physicians to be more deliberate and strategic about their use of diagnostic reasoning strategies before a clinical reasoning task and more critically reflective on those strategies after the task may be an important way to improve performance in these types of clinical reasoning exercises.

In our study, it was common for students to reference strategies related to test-taking tactics, or the mechanistic approach to an MCQ (e.g., reading the question or answers first vs reading the vignette first) in addition to or instead of a diagnostic reasoning strategy. This may indicate that students view success with these types of questions as arising from choosing the right test-taking tactic rather than applying the right diagnostic strategy. This presents an opportunity for educators working with students who seek to improve their test taking performance: namely, to reframe the MCQ as a diagnostic reasoning exercise. While there may be different levels of success associated with different mechanistic approaches to the question (top-to-bottom approach vs reading the question and/or answers first), these approaches have limited utility in clinical practice, where there is no list of answer choices provided with each patient. For students seeking to improve their performance, less emphasis on test-taking tactics and more on strategies to enhance the diagnostic reasoning process may benefit both test-taking performance and clinical practice.

Cognitive flexibility, or the ability to change or reframe a perspective, has been positively associated with high achievement during college and medical school as well as lower rates of burnout [24,25,26]. In our study, we found that most students tended to maintain their strategy even after making a mistake and getting a question wrong. This may be due to the influence of prior success with a particular strategy or the effect of different preclerkship curricular experiences. Nevertheless, teaching a variety of diagnostic strategies and encouraging learners to be flexible in their approach to clinical reasoning, especially in response to failure, may improve student performance and give students a variety of techniques that they can apply in clinical practice.

Our study’s strengths include its multicenter nature, the exploration of subprocesses in all three phases of SRL for a previously unexplored task of MCQs, and multiple iterations that allowed us to examine changes in strategy over the course of seven clinical vignette-based practice questions. As students spend a large proportion of their study time on these practice questions and as they are a common tool for assessing medical knowledge, it is important for medical educators to understand the cognitive and regulatory processes inherent to these questions. We have described patterns of strategic planning, metacognition, and attribution that may be maladaptive, such as the lack of explicit endorsement of diagnostic reasoning strategies as a focal point and lack of reflection on these strategies as reasons for failure, despite utilization of these strategies as evidenced by metacognitive inquiry, as well as a large proportion of negative thoughts about knowledge and abilities. These results are important for medical educators to consider as they seek to improve the regulatory processes and performance of poor test takers. Nevertheless, our study has limitations. Participants on average performed around the 28^th^ national percentile on the NBME SEM and so results may not generalize to very low performers who may be most in need of improvement in their approach to practice questions. We did not further subcategorize management reasoning into task-specific or task-general processes, nor did we examine the effect of different types of MCQs which may be areas for future exploration and research. Our study was conducted during the COVID-19 pandemic which affected the educational experience of participants and may have affected our results. Finally, our study was not powered to examine associations between SRL subprocesses and academic outcomes.

Our study adds to the growing body of literature that uses SRL microanalysis to explore regulatory processes in medical learners engaged in a variety of academic and clinical tasks [17,18,19,21,27]. In this work we describe in detail the strategies, metacognition, attributions, and adaptations of students as they engage in answering clinical vignette-style practice questions. Based on our results medical educators may wish to emphasize the explicit identification of diagnostic reasoning processes as a strategic focus and critical evaluation of these diagnostic processes to improve performance. Additional studies are needed to examine the correlations between SRL subprocesses and academic outcomes and examine the subprocesses of very low performing students to determine areas for improvement.

## Additional File

The additional file for this article can be found as follows:

10.5334/pme.833.s1 Supplement. The additional file includes the Question Review Form, a description of the SRL Microanalytic Protocol, and the SRL Microanalytic Protocol Coding Rubric.
