# Supplementary material for: Using Self-Regulated Learning Microanalysis to Examine Regulatory Processes in Clerkship Students Engaged in Practice Questions
Source: Perspect Med Educ. 2023 Oct 13;12(1):385–98. doi: 10.5334/pme.833 (PMC10573650; doi:10.5334/pme.833)
Supplement: Supplement. — The additional file includes the Question Review Form, a description of the SRL Microanalytic Protocol, and the SRL Microanalytic Protocol Coding Rubric. [file pme-12-1-833-s1.pdf]

Supplement

SRL Microanalytic Protocol – pp. 2-4

Coding Scheme for Microanalytic Protocol – pp. 5-15

Scoring Scheme for Microanalytic Protocol – pp. 16-19

PRINT SINGLE SIDED

Participant Name:____

Study ID Number:__________ (office use only) Date:___________ Investigator:____________

Site: USU GU UCF

*****START AUDIO RECORDING*****

FOR INTERVENTION PARTICIPANTS ONLY, PLEASE BEGIN BY ASKING THE FOLLOWING:

"Before we begin, I would like to get your general impression of the method for answering test questions that we showed you in the last meeting. Did you find the method helpful?

"Why or why not?"

"Is there anything you would change about the method?"

FOR ALL PARTICIPANTS

Introduction and Warm-Up

"Thank you for participating in this study. During the next 60-90 minutes, I will give you a series of practice questions and ask you to read out loud and think out loud as you go through the question. I want you to approach each question and work through it exactly as you usually do but the only difference is I want you to read out loud and think out loud as you go. I will be asking you some additional questions as you work through the test question.

We are going to do a warm up activity now to acquaint you with the process. I am going to ask you to think aloud as you work through some practice questions. What I mean by think aloud is that *I want you to say your thoughts out loud from the moment you start reading the practice question until your final answer. Say as much as you feel comfortable saying. Don't try to plan or explain what you say, just act as if you were speaking to yourself.* If you are silent for a long time, I'll ask you to think aloud, and at certain points I will ask you questions about your thought process. I am more interested in the description of your thought process than in whether you get the question right or wrong. Do you have any questions about what I am asking you to do?

Here is the warm up question to help you practice thinking aloud.

1. **Name 5 items you might find in a museum.**
   (Any other thoughts while you were answering this questions? I want to hear those thoughts too but don't try to explain them, just think aloud.) [Reinforce what was done right.]

2. **What is the 6th letter after C?**
   We don't want a summary, we want to hear your thoughts as you think through your answer. [Correct answer is "I". Reinforce what was done right.]

Now we will begin the study questions.

[DID YOU START AUDIO RECORDING??]

PRACTICE QUESTION #1

FORETHOUGHT PHASE
Give student test question #1. All information is visible (no covering of answer, etc).

Interview Question #1 – Strategic Planning
“Here is the first practice question. Before you begin, I have a question for you. What are your plans for how to approach this test question in order to answer it correctly?”
Pause to allow student to reflect/answer. This gets repetitive after a few questions, but please keep asking. Will be interested to see if/how strategy changes over the series of questions.

“**Can you be more specific?”** (Ask this if student gives an overly general answer, e.g., “I’m going to use the strategy on the Question Review Form.”)

PERFORMANCE PHASE
“Thank you for your answer. Now you may proceed with the read aloud and think aloud of the practice question.”
(Student begins to work through the practice question. Remind the student to read/think out loud if needed.)

(After the student has reached the end of the stem…)

Q2 – Metacognitive Monitoring
“Let me briefly pause you to ask a few additional questions. What have you been thinking about as you have been working through this question?”
Pause to allow student to reflect/answer.

“Is there anything else?”
Pause to allow student to reflect/answer.

Q3 – Quality of Disease Scripts/Script Recognition
“What is the most likely diagnosis for this patient?”
Pause to allow student to reflect/answer. [Note: give student ample time to try and come up with a diagnosis. If the student is truly unable to come up with a diagnosis, you can skip ahead to Q7, top of next page.]

Q4– Quality of Disease Scripts/Script Specificity
“Please tell me how you could make your leading diagnosis more specific. For example, how would you refine the diagnosis in terms of the specific clinical scenario or level of severity depicted in this clinical vignette?” You can skip this if the student’s answer to Q3 is already appropriately specific to the clinical scenario. Teacher can give examples of subtypes of disease not related to the question at hand, e.g., different types of depression, different levels of severity for community-acquired pneumonia.
Pause to allow student to reflect/answer.

Q5-6 – Quality of Disease Scripts
"What features of this clinical vignette support your diagnosis?"
Pause to allow student to reflect/answer.

"What features of the clinical vignette do NOT support your diagnosis? There might not be anything, but please consider whether there is some feature that doesn't fit."
Pause to allow student to reflect/answer.

"Thank you. Please continue with the practice question."

After the student selects their answer and marks it on the answer sheet…

Q7 - Self-Evaluation
"How confident are you that your answer will be correct?" (Student should circle their level of confidence on the answer sheet)

REFLECTION PHASE
"Now I will tell you the correct answer. The correct answer is…"

Q8 - Causal Attribution
"Your answer was [correct/incorrect]. What do you think is the number one reason why you got this particular question right (or wrong)?"
Pause to allow student to answer.

"Are there any other reasons why you got this particular question right (or wrong)? Please tell me about these reasons."
Pause to allow student to reflect/answer.

"Is there anything else you need to learn from this question?"

Q9 - Adaptive Inferences
"What should you do in order to get this question or a similar question right in the future?"

REPEAT PROCESS FOR PRACTICE QUESTIONS #2-7.

****END AUDIO RECORDING*****

Please remind student not to share information about the study with other students.

POST-ENCOUNTER ASSESSMENT BY FACULTY

1. Did the student cover answers before reading the question? ___Yes ___No

2. Did the student predict an answer before looking at the answer choices? ___Yes ___No

SRL Microanalytic Protocol for Clinical Vignette-Style Multiple Choice Questions

Coding Rubric

Diagnostic Reasoning Process – List of diagnostic reasoning process/tactics

a1. Identify symptoms/signs – responses indicating a focus on identifying any of the presenting symptoms/signs. This response **DOES NOT** include attempts to identify pertinent symptoms/signs or to rank symptoms/signs in terms of importance or to relate symptoms/signs to a diagnosis.

"To figure out all of the things that are wrong with the patient"; "To identify the presenting symptoms"; "To figure out the problem list"; "She has tenderness with rebound and decreased bowel sounds"

a2. Identify contextual/environmental factors – responses involving a focus on the demographic characteristics of the patient, background variables, timeline of illness, or historical factors (does not pertain to symptoms/signs). This response **DOES NOT** include attempts to identify pertinent symptoms/signs or to rank symptoms/signs in terms of importance or to relate symptoms/signs to a diagnosis.

"I need to consider and look at the person's ethnicity and family history"; "the paitent is young and the age of people that get Hodgkin's lymphoma," "the patient's history of high cholesterol"

a3. Prioritize relevant/pertinent symptoms/signs or features – responses pertaining to attempts to identify a small set of symptoms/signs/features that one needs to focus on; symptoms/signs/features that appear to be most **relevant, important, or valuable**. Responses **NOT** included in this category would be prioritizing symptoms/signs with a focus on developing a diagnosis.

"To identify a problem list…list out the most important ones "; "Vomiting and stomach cramps seem to be the most important…so I need to focus on them"; "I don't seem to be able to figure out which of the symptoms/signs is most important"; "Trying to rule out what I don't think is important"; "To focus more on <symptoms or sign X>"

a4. Integration – responses signifying synthesis and considering how symptoms/signs, historical factors, patient demographics etc fit together to arrive at a diagnosis. This category includes comparing and contrasting symptoms/signs, developing hypotheses, and responses referencing etiology of disease. The student **MUST** mention the word symptoms/signs, problem list or present actual case symptoms/sign or contextual factors (at least one) to be counted. Do not code a1, a2, a5, or e when coding a4.

a4a. Implicit integration: student lists features and a diagnosis, but lacks a clearly integrative statement or word.

"Acute decompensated liver failure, that he's had a history of HE, he's disoriented, somnolent, high ammonia levels."

a4b. Explicit integration:student makes explicit their attempts to integrate symptoms to arrive at a diagnosis using words/phrases like "Fits together with," "supports," "because," "so," "but," "given," "therefore," "connects," etc.

"My goal is to figure out how the symptoms/signs connect"; "Vomiting, fever, and frequent urination seem to fit together to support a diagnosis of …"; "I'm looking for a diagnosis to cover all the symptoms/signs"; "I'm not completely sure about all the signs and symptoms/signs that would be expected with the diseases I thought of"; "List a differential diagnosis that will cover everything on the problem list"; "Figure out how <symptoms or sign X> relates to <symptoms/signs Y>"; "I don't know what can cause this combination of symptoms/signs"; "Trying to figure out if it's like acute prostatitis, which it seems like it because the prostate was tender, but he didn't respond to ciprofloxacin, so that's weird."

a5. Comparing/contrasting diagnoses – responses in which individuals consider different diagnoses in their pursuit of an eventual diagnosis. The student **MUST** mention multiple diagnoses to be scored for this category. Attempts to arrive at a differential diagnosis.

“It could be diabetes but it might also be anemia”: “I need to look at all of the factors to develop several diagnoses and then pick the best one”

a6. General/Other – responses that specifically refer to a general method or procedure to follow. This category **DOES NOT** include responses that can be coded as a specific strategy or tactic (1-5).

“To do all of the rights steps to solve it”; “To focus on the procedure that we learned”; “To integrate all information”; “To see what is important”; “The diagnosis” (or a specific Dx, like “diabetes mellitus”)

Strategic Planning

“What are your plans for how to approach this test question in order to answer it correctly?”

Coding Categories

*Note: when a participant gives a response that indicates their strategy is unchanged from the prior question, code the present response the same as the prior response.

(a) Diagnostic reasoning sub-processes – responses pertaining to specific sub-processes of the diagnostic reasoning process **(see page 2 – e.g., identify symptoms/signs, prioritize symptoms/signs, integration of symptoms/signs; comparing and contrasting diagnoses)**

(b) Management reasoning process – responses reflecting the need to identify the management steps for identifying/selecting the next best diagnostic test to order or therapy to initiate.

"The standard of care for treating this condition is ..." "According to the ACC/AHA guidelines, the recommended treatment is..."

(c) Test-taking tactics– responses referencing methods, approaches, or procedures for understanding and completing the question. These responses pertain primarily to actions for working through the test question.

c1) Reading the clinical vignette first and/or figure out what is wrong with the patient first (top-to-bottom approach)

“I guess read it, try to figure out a differential.. and answer it without using the options, if I can.”

“Probably read through and try to get a diagnosis…and then look at the question answers.”

c2) reading the prompt (question) first

“Maybe this time I'll read the question first... [read] the end question before reading the stem.”

“I start with the question.”

c3) reading the answers first

“First thing I look at is the answer options.”

c4) process of elimination (eliminate wrong answers)

cx) Test-taking tactic not clearly identified above

“Well, I like to look at what the question's actually asking at some point. That's all I got.”

“Ruling things out worked for me last time so maybe I'll do that again.”

(d) Personal preferences – responses may indicated the participant is relying on his/her own intuition, biases, or gut instinct.

“I just use my gut and instincts”

“I will just do whatever seems to be right based on the case”

(e) Outcome oriented: responses pertaining getting to the right answer, irrespective of the processes above.

“Just figuring out what the diagnosis is.” “Just get to the right answer.”

(f) Prior experience: Rely on prior experience with patients/cases to answer the question.

“apply any cases I've seen before”

(g) N/A

(h) N/A

(i) N/A

(j) N/A

(k) N/A

(l) N/A

(m) Don’t know/none – responses conveying that the student does not have a plan or is not really aware of one.

“I really don’t know”; “Nothing really…just do my best”

(n) Other – responses that do not fit into any of the categories a-m.

Metacognitive monitoring

"What have you been thinking about or focusing on as you have been working through this question?"

Coding Categories

(a) Diagnostic reasoning sub-processes – responses pertaining to specific sub-processes of the diagnostic reasoning process **(see page 2 – e.g., identify symptoms/signs, prioritize symptoms/signs, integration of symptoms/signs; comparing and contrasting diagnoses)**

(b) Management reasoning process – responses reflecting the need to identify the management steps for identifying/selecting the next best diagnostic test to order or therapy to initiate.

"The standard of care for treating this condition is ..." "According to the ACC/AHA guidelines, the recommended treatment is..."

(c) Test-taking techniques - responses referencing methods, approaches, or procedures for understanding and completing the question. These responses pertain primarily to actions for working through the test question.

c1.) Reading the clinical vignette first, figure out what is wrong with the patient first (top-to-bottom approach)

"I'm thinking about reading through the clinical vignette and arriving at the diagnosis before looking at the answer choices."

c2.) reading the prompt (question) first

"I'm thinking about reading the question first and then the clinical vignette."

c3) reading answers first

"I'm thinking about the answer choices first."

c4) process of elimination

"I'm ruling out answers as I go."

cx) other test taking technique/strategy that can't be used in clinica practice/only works due to the nature of these MCQs (often using the answers as a crutch)

"I think being able to recognize, specifically in practice question land, but what is stable versus unstable."

(d) Personal preferences – responses may indicated the participant is relying on his/her own intuition, biases, or gut instinct. This response does not references specific clinical features or the weight given to these features but mentions or references intuition, preferences, biases more broadly.

"I'm thinking about my gut instinct for this case."

(e) Name of Diagnosis – responses pertaining to the name of a single diagnosis or condition that the patient potentially exhibits. The response could reflect a desire to know what the condition is or reference to one specific diagnosis.

"Just figuring out what the diagnosis is."

"Stable angina is my primary diagnosis."

"**I was just thinking what type of infection does she have?**

(f) Prior experience: References to prior experience with a patient (a positive, negative, or neutral).

“I was thinking about a patient I had who in which we discussed hepatic encephalopathy as one of the possible things that we had to rule out.”

(g) Case difficulty - responses pertaining to the difficulty level of the case. To be scored in this category, the response **CANNOT** involve statements pertaining to the students’ knowledge or ability. If a student gives a case difficulty response AND an ability response, just score the total response as ability.

“This case is confusing. It is not clear what is the key thing to focus on”; “It was obvious what the diagnosis was…very easy case”

(h) Ability/knowledge – statements referring to one’s ability, knowledge, or efficacy to come up with an appropriate diagnosis.

h1) Positive references to one’s own knowledge or abilities or efficacy.

h2) Negative references to one’s knowledge or abilities or efficacy.

“I was never that good at diagnosing”; “I am not confident that I can do this case”; “I need to practice more…I am not very good at this.”

h3) Neutral references to one’s own knowledge or abilities or efficacy.

(i) N/A

(j) Lack of case information – responses pertaining to a perceived lack of data or information provided about the case.

“I do not think all of the key information is provided”; “I need to learn more about the patient’s background and history”

(k) N/A

(l) Interest or importance of topic or case –responses reflecting perceptions regarding the interest, relevance, importance, or value of the particular case

“I just find this case to be so interesting”; “This is a very important topic that I need to study more”

(m) Don’t know/none – responses conveying that the student is not really focusing on anything in particular or is not aware of his/her thinking. This is only score if no other response is given.

“I really don’t know”; “Nothing really…just do my best”

(n) Other – responses that do not fit into any of the categories a-m.

Causal Attributions

“What do you think is the number one reason why you got this particular question right/wrong?”

Coding Categories

(a) Diagnostic reasoning sub-processes – responses pertaining to specific sub-processes of the diagnostic reasoning process **(see page 2 – e.g., identify symptoms/signs, prioritize symptoms/signs, integration of symptoms/signs; comparing and contrasting diagnoses)**

(b) Management reasoning process – responses reflecting process for identifying/selecting the next best diagnostic test to order or therapy to initiate.

“I had a lot of difficulty figuring out the next most appropriate test to administer”;

(c) Test-taking techniques: responses referencing methods, approaches, or procedures for understanding and completing the question. These responses pertain primarily to actions for working through the test question.

c1.) Reading the clinical vignette first, figure out what is wrong with the patient first (top-to-bottom approach)

“I focused on reading the case first and figured out for myself what was going on before looking at the answers.”

c2.) reading the prompt (question) first

“I read the question first and got off track.”

“I read the question first and focused on treatment options for this case.”

c3) reading answers first

“I read the answers first and that gave me and idea of what to focus on in the case.”

c4) process of elimination:

“I was able to rule out two of four [answer choices].”

cx) other test-taking technique/artificial strategy that only works due to the nature of these MCQs (often times using answers as a crutch)

“be able to distinguish between the answers what's more pressing or what's the priority.”

“I think being able to recognize, specifically in practice question land, but what is stable versus unstable.”

(d) Personal preferences – responses may indicated the participant is relying on his/her own intuition, biases, or gut instinct. This response does not references specific clinical features or the weight given to these features but mentions or references intuition, preferences, biases more broadly.

“**I guess just clinical intuition. She seems pretty sick. I would admit her to the hospital”**

"I generally have maybe a bias to any answer that says increase the dosage of something.”

(e) N/A

(f) Prior experience: Reference to knowledge gained by prior experience with similar patients or paper cases/practice questions or study aids. References to inherent knowledge (“I knew this, didn’t know that”) should be coded (h)

f1) positive (this prior experience helped)

“experience in the clinic with patients coming back after already being on a lot of meds like that”

“a lot of it too is just like repetition and seeing vignettes on U-World and my patients in the hospital”

f2) Negative (this prior experience or lack thereof was detrimental).

“I think part of it is that I haven't actually seen any questions like this in a long time.”

“That’s not what we did with the patient we had on the wards.”

(g) Case difficulty - responses pertaining to the difficulty level of the case. To be scored in this category, the response **CANNOT** involve statements pertaining to the students’ knowledge or ability. If a student gives a case difficulty response AND an ability response, just score the total response as ability.

“This case is confusing. It is not clear what is the key thing to focus on”; “It was obvious what the diagnosis was…very easy case”

(h) Ability/knowledge – statements referring to one’s ability, knowledge, or efficacy to come up with an appropriate diagnosis.

h1) Positive reference to ability/knowledge (I had the knowledge)

“I knew the diagnosis.” “I know a lot about this condition, so it was easy”

h2) Negative reference to ability/knowledge (it was lacking)

“I was never that good at diagnosing”

h3) lack of confidence in one’s knowledge, overthinking, second-guessing

“I am not confident that I can do this case,” “I think that I was overthinking my answers too much”

(i) Luck – statements pertaining to luck or chance that the correct answer was achieved.

“I took a guess and I got it correct” “It was 50/50”

(j) N/A

(k) Focus/effort: Statements referencing successful ability to focus/not get distracted/not overlook things

“Not getting distracted by all of the extra information.”

“I didn't let myself get distracted by a couple things that I wasn't sure of.”

(l) N/A

(m) Don’t know/none – responses conveying that the student does not give an attribution or is not aware of one.

“I really don’t know”; “Nothing really…just do my best”

(n) Other – responses that do not fit into any of the categories a-h.

“I’m missing something” (as this doesn’t indicate whether it’s knowledge they’re missing [poor knowledge] or if the case is missing information [task difficulty])

o) Subjective nature of the question:

o1)Question was incorrect/unfair/defective: Statements referencing a perceived defect in the question as the reason the learner arrived at the answer they did. This is not a statement referencing a difficult question but a statement referencing an unfair or erroneous question (as perceived by the learner).

“I think that's baloney because rebound tenderness is like classic what they teach you for surgical abdomen.”

o2) Question was an easy kind of question: **“I think it's also helpful that it's a diagnostic type question. I feel like what's the next best test to order is a much easier question than what's the next best step in treatment?”**

Adaptive Inferences

“What should you do in order to get this question or a similar question right in the future?”

Coding Categories

(a) Diagnostic reasoning sub-processes – responses pertaining to specific sub-processes of the diagnostic reasoning process **(see page 2 – e.g., identify symptoms/signs, prioritize symptoms/signs, integration of symptoms/signs; comparing and contrasting diagnoses)**

(b) Management reasoning process – responses reflecting process for identifying/selecting the next best diagnostic test to order or therapy to initiate.

“I need to consider the level of severity when I come up with my treatment plan.”

(c) Test-taking techniques: Statements referencing the student’s approach to the test question.

c1) Reading the clinical vignette first, figure out what is wrong with the patient first (top-to-bottom approach)

“I should focus on reading the case first and figure out for myself what was going on before looking at the answers.”

c2) reading the prompt (question) first

“Read the question first next time.”

c3) reading answers first

“Read the answers first next time.”

c4) process of elimination:

“I was able to rule out two of four [answer choices].”

cx) other test-taking technique/artificial strategy that only works due to the nature of these MCQs (often times using answers as a crutch)

“be able to distinguish between the answers what's more pressing or what's the priority.”

“I think being able to recognize, specifically in practice question land, but what is stable versus unstable.”

(d) Personal preferences/gut/intuition: Personal preferences/intuition: Statements referencing the need to trust one’s intuition or gut instinct or first reaction.

“Go with my gut.”

(e) N/A

(f) N/A (code increase experience/exposure as increase knowledge/ability below)

(g) N/A

(h) Increase knowledge/ability: Statements referring to students attempt to perform some correct or remedial actions to enhance knowledge or skill for a future task.

h1) Gain more knowledge by reading/looking things up/reviewing/studying

“I guess, look over C. diff complications and indications for surgery.”

h2) Gain knowledge by more experience/exposure to these types of patients in clinic or hospital

“I need to see more patients with COPD in clinic.”

h3) Gain more knowledge by doing more practice question on this topic

"Do more practice questions on C diff."

h4) Gain more knowledge by an interactive method other than read more, e.g., write a paragraph, create a chart, create a flashcard, make a chalk talk, teach my cat, etc.

"Maybe start writing out the protocols for disease processes, like what's step one, two, three, etc."

hx) Gain more knowledge by method not specified.

"Know the flow chart"

"Work on smears and paying attention to hemo stuff"

"Just basic EKG reading stuff."

(i) N/A

(j) N/A

(k) Focus/effort: Statements referencing ability or need to focus/pay/attention/not get distracted/not overlook things

"Pay attention to when they give you the long list of symptoms."

(l) N/A

(m) Don't know/none – responses conveying that the student does not have a goal or plan or is not really aware of one.

"I really don't know"; "Nothing really…just do my best"; "Just keep doing what I'm doing"

(n) Other: Responses that do not fit into any of the categories a-m.

"Study harder" (as this doesn't indicate whether they mean study the case harder [volition] or study harder during/outside of class [non-task/poor strategies])

"Remember something I've forgotten"

Scoring Template Final – SRL and Test-taking Study

(Examples of responses for each category are available in the Coding Rubric)

Strategic planning

a) Diagnostic reasoning process
1. Identify symptoms: +1

2. Identify contextual/environmental factors: +1

3. Identify or prioritize relevant/pertinent symptoms or features: +2

4. Integration: +3

5. Comparing/contrasting diagnoses: +3

6. General/Other: +0

b) Management reasoning process: +1

c) Test-taking tactics

c1) Reading vignette first: +1
c2) Reading prompt first: +0
c3) Reading answers first: -1
c4) process of elimination: +0 - neutral
cx) other test taking tactic: +0 – neutral

d) Personal preferences: -1
e) Outcome oriented: +0
f) Prior experience: +0
g) N/A for this subprocess
h) N/A
i) N/A
j) N/A
k) N/A
l) N/A
m) Don't know/none:

m1) Don't know plus another theme: +0
m2) Don't know as the only response: -2

n) Other: +0

<u>Metacognitive monitoring</u>

a) Diagnostic reasoning process

1. <u>Identify symptoms</u>: +1

2. <u>Identify contextual/environmental factors</u>: +1

3. <u>Identify or prioritize relevant/pertinent symptoms or features</u>: +2

4. <u>Integration</u>: +3

5. <u>Comparing/contrasting diagnoses</u>: +3

6. <u>General/Other</u>: +0

b) Management reasoning process: +1

c) Test-taking tactics

- c1) Reading vignette first: +1
- c2) Reading prompt first: +0
- c3) Reading answers first: -1
- c4) process of elimination: +0
- cx) other test taking tactic: +0

d) Personal preferences (i.e., non-task theme): -1

e) Outcome oriented: +0

f) Prior experience: +0

g) Case difficulty: -1

h) Ability/knowledge:

- h1) positive reference: +0
- h2) negative reference: -2
- h3) neutral reference: +0

i) N/A for this subprocess

j) Lack of case information: -1 (analogous to case difficulty)

k) N/A for this subprocess

l) Interest or importance of topic or case: -1

m) Don't know/none:

- m1) Don't know plus another theme: +0
- m2) Don't know as the only response: -2

n) Other: +0

<u>Causal Attribution</u>

a) Diagnostic reasoning process
1. <u>Identify symptoms</u>: +1

2. <u>Identify contextual/environmental factors</u>: +1

3. <u>Identify or prioritize relevant/pertinent symptoms or features</u>: +2

4. <u>Integration</u>: +3

5. <u>Comparing/contrasting diagnoses</u>: +3

6. <u>General/Other</u>: +0

b) Management reasoning process: +1
c) Test-taking tactics
- c1) Reading vignette first: +1
- c2) Reading prompt first: +0
- c3) Reading answers first: -1
- c4) process of elimination: +0
- cx) other test taking tactic: +0

d) Personal preferences (i.e., non-task theme): -1
e) N/A for this suprocess
f) Prior experience: +0
g) Case difficulty: -1
h) Ability/knowledge:
- h1) positive reference: +0
- h2) negative reference: +0 (as identifying what one didn't know is not maladaptive here)
- h3) neutral reference: +0

i) Luck: -1
j) N/A for this subprocess
k) Focus/effort: +1
l) N/A for this subprocess
m) Don't know/none:
- m1) Don't know plus another theme: +0
- m2) Don't know as the only response: -2

n) Other: +0
o) Question itself
- o1) Question was unfair: -1 (analogous to difficulty)
- o2) Question was an easy kind of question: +0

<u>Adaptive Inferences</u>

a) Diagnostic reasoning process

1. <u>Identify symptoms</u>: +1

2. <u>Identify contextual/environmental factors</u>: +1

3. <u>Identify or prioritize relevant/pertinent symptoms or features</u>: +2

4. <u>Integration</u>: +3

5. <u>Comparing/contrasting diagnoses</u>: +3

6. <u>General/Other</u>: +0

b) Management reasoning process: +1

c) Test-taking tactics

- c1) Reading vignette first: +1
- c2) Reading prompt first: +0
- c3) Reading answers first: -1
- c4) process of elimination: +0
- cx) other test taking tactic: +0

d) Personal preferences (i.e., non-task theme): -1

e) N/A for this subprocess

f) N/A for this subprocess

g) Case difficulty: -1

h) N/A for this code

i) N/A for this subprocess

j) N/A for this subprocess

k) Focus/effort: +1 (volitional/self-control)

l) N/A for this subprocess

m) Don't know/none:

- m1) Don't know plus another theme: +0
- m2) Don't know as the only response: -2

n) Other: +0

o) N/A for this subprocess

p) Increase knowledge

- p1) By reading/looking things up/reviewing: +0
- p2) by more exposure to these types of patients in clinic/hospital: +1
- p3) by doing more practice questions on this topic: +1
- p4) by an interactive method other than read more: +2
- px) by method not specified: +0
